# Efficacy and exploratory treatment-response modeling of acupuncture for acute migraine based on DIA proteomics: a study protocol for a randomized controlled trial

**DOI:** 10.3389/fmed.2026.1939184

**Published:** 2026-09-21

**Authors:** Qiang Zhang, Miao Liu, Fu Xu, Hailun Jiang

**Affiliations:** 1 BeiJing Daxing District Hospital of Integrated Chinese and Western Medicine, BeiJing, China; 2 The First Affiliated Hospital of Zhejiang Chinese Medical University (Zhejiang Provincial Hospital of Traditional Chinese Medicine), Hangzhou, China

**Keywords:** acupuncture, migraine, proteomics, protocol, response

## Abstract

**Background:**

According to estimates from the 2019 Global Burden of Disease (GBD) study, approximately 1.1 billion people worldwide suffer from migraine, with a prevalence rate of about 14% to 15%. Despite the development of various medications for different types of migraine, such as acetaminophen and caffeine combination preparations, most patients still experience suboptimal symptom control. Therefore, identifying effective and safer alternative treatments holds significant clinical importance. Acupuncture was formally recommended by the World Health Organization as a migraine therapy as early as 1979. In recent years, substantial domestic and international research evidence has demonstrated the distinct advantages of acupuncture in treating migraine. Proteomics technology can assess the final products of adaptive changes in the body following acupuncture, thereby establishing a close association with disease progression.

**Objective:**

This study aims to evaluate the efficacy of a single acupuncture session for acute migraine, identify subgroups with different short-term treatment responses, and explore baseline plasma protein biomarkers potentially associated with acupuncture response. An exploratory proof-of-concept model will be developed to assess whether a small set of prespecified clinical and proteomic features may contain preliminary predictive information regarding short-term treatment response.

**Methods:**

This study is divided into three parts. First, this randomized controlled trial will recruit 86 acute migraine patients. All participants will be randomly assigned in a 1:1 ratio to either the real acupuncture (RA) group or the sham acupuncture (SA) group. Each patient will receive one acupuncture treatment session. The primary outcome is the between-group difference in the change in visual analog scale (VAS) scores from baseline to 24 h post-treatment. Secondary outcomes include assessment of VAS scores at 72 h and 1 week post-needling; Hamilton Anxiety Scale/Hamilton Depression Scale (HAMA/HAMD) at 1 week post-needling; clinical response rates at 24 h, 72 h, and 1 week post-needling; and migraine attack count, attack frequency, and attack duration at 24 h, 72 h, and 1 week post-needling. Second, using the prespecified percentage change in VAS as the efficacy metric, 20 participants will be selected from the 43 participants in the RA group, including 10 high-responders and 10 low-responders. Baseline plasma samples collected before acupuncture will be analyzed using DIA-based proteomics to identify differentially expressed proteins and explore biological pathways potentially associated with short-term acupuncture response. Third, an exploratory proof-of-concept treatment-response model will be developed using the clinical and proteomic data from these 20 participants. Because of the limited sample size, no training-test split will be performed and complex machine-learning algorithms will not be compared. A parsimonious penalized logistic regression model will be assessed using nested leave-one-out cross-validation, with all data-dependent preprocessing and feature-selection procedures performed within each resampling iteration. The results will be considered hypothesis-generating and will require independent external validation.

**Conclusion:**

This protocol describes a randomized, single-blind trial that will evaluate the efficacy of acupuncture for acute migraine and explore plasma protein biomarkers potentially associated with short-term acupuncture response. The exploratory model is intended to assess methodological feasibility and generate hypotheses rather than provide a validated clinical decision-support tool.

**Clinical Trial Registration:**

www.chictr.org.cn, identifier ITMCTR2026000359.

## 1 Introduction

Migraine is a common clinical disorder of the central nervous system, with moderate to severe unilateral throbbing headache as the main clinical manifestation, often accompanied by photophobia, phobia, nausea, vomiting and other symptoms. Approximately two-thirds of migraine cases are classified as migraine without aura (1). Migraine has a significant genetic predisposition, with a heritability of 40% to 60%. The risk of migraine in the offspring generation is 1.5 to 4 times higher than that of the general population (2). Migraine is a major global public health issue characterized by high prevalence, significant disability, and substantial socioeconomic burden. Currently, Western medical treatments primarily target single pathways, with efficacy that remains suboptimal (2, 3). Additionally, approximately 40% of patients develop medication-overuse headaches (MOH), which can easily perpetuate a vicious cycle of headache (4, 5).

Acupuncture therapy can be used both as acute treatment and as preventive therapy (6). Previous studies suggested acupuncture was no more effective than sham acupuncture for migraine (7). However, multiple recent Chinese studies have confirmed that acupuncture treatment significantly reduces the frequency of attacks in episodic migraine patients. Compared to sham acupuncture and placebo controls, acupuncture demonstrated faster onset of action, greater effect size, and more sustained efficacy (8–10). Currently, the mechanism underlying acupuncture treatment for migraine remains unclear. Due to individual variations in migraine frequency, characteristics, and severity, there is a lack of predictive biomarkers for treatment efficacy and side effects. Personalized treatment often requires repeated validation, which increases additional medical costs and may delay patients’ access to effective therapy. Therefore, there is a clinical need for a method to predict patient response to acupuncture, enabling prospective adjustment of treatment protocols for different individuals.

Omics technologies have advanced rapidly over the past few decades, becoming a key approach for discovering biomarkers in neurological disorders, immune system diseases, and cardiovascular conditions. Different omics methods correspond to distinct levels of biological information. Taking proteomics as an example, it assesses the end products of adaptive changes in the body by conducting large-scale analyses of the entire proteome at specific time points, thereby establishing strong correlations with disease progression. The value of this technology in clinical migraine research is mainly reflected in two aspects at present. First, by comparing protein expression profiles between acute migraine patients and healthy individuals, differentially expressed proteins can be identified, which can not only deepen our understanding of pathological mechanisms but also reveal novel therapeutic targets. Second, tracking protein dynamics before and after treatment enables assessment of therapeutic efficacy and short-term acupuncture response. Therefore, exploring the clinical efficacy and mechanisms of acupuncture in treating acute migraine through proteomics holds fundamental and clinical significance.

A treatment-response prediction model is a mathematical tool that integrates baseline clinical characteristics and biomarkers to estimate an individual patient's likelihood or degree of response to a specific treatment. Once adequately developed and externally validated, such models may support the early identification of patients who are more likely to benefit from treatment and facilitate the optimization of therapeutic strategies. In the present study, baseline clinical characteristics and candidate protein features identified through within-fold differential protein screening will be integrated to develop an exploratory proof-of-concept model of short-term acupuncture response in patients with acute migraine. The analysis aims to prioritize candidate baseline biomarkers potentially associated with treatment response and to generate preliminary evidence for future model development and validation in larger independent cohorts. Given the limited sample size, the resulting model will be considered hypothesis-generating and will not be used to guide personalized clinical decision-making at this stage.

Accordingly, this study aims to identify subgroups with varying responses to acupuncture treatment for acute migraine, comprehensively analyze their shared clinical characteristics, and identify key plasma protein biomarkers. Based on these findings, an exploratory proof-of-concept model will be developed.

## 2 Methods and design

### 2.1 Study Ⅰ: differences in clinical efficacy of acupuncture intervention for acute migraine

#### 2.1.1 Study design

This is a randomized, single-blind, sham-controlled study. This protocol is under the Standard Protocol Items: Recommendations for Intervention Trials (SPIRIT) guidelines. Figure 1 provides the detailed information of the study design.

**Figure 1:**
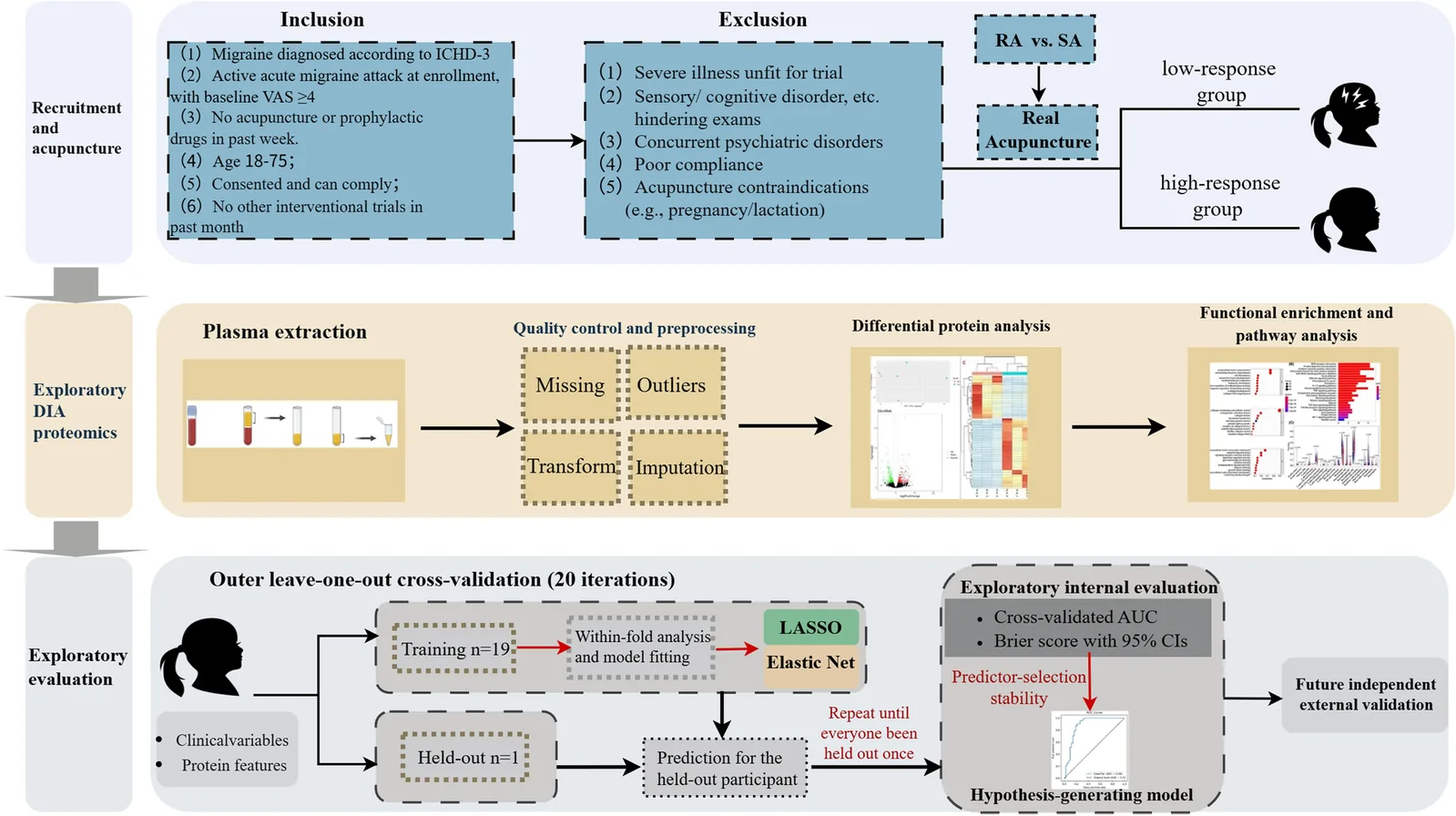
Study design and exploratory treatment-response modeling workflow. A total of 86 participants with an active acute migraine attack will be randomly assigned to the real acupuncture or sham acupuncture group in a 1:1 ratio. Twenty participants from the real acupuncture group, comprising 10 high-responders and 10 low-responders according to the prespecified percentage reduction in VAS at 24 h, will undergo baseline plasma DIA proteomic analysis. An exploratory penalized logistic regression model will be evaluated using nested leave-one-out cross-validation. All data-dependent preprocessing, feature selection, and hyperparameter tuning will be performed within each training fold to minimize information leakage. Model performance and predictor stability will be interpreted as hypothesis-generating, and independent external validation will be required before clinical application.

#### 2.1.2 Study participants

##### 2.1.2.1 Recruitment strategy

The study will be carried out from January 2026 to January 2028 at the Zhejiang Provincial Hospital of Traditional Chinese Medicine. Acute migraine patients will be recruited from the outpatient and inpatient departments.

##### 2.1.2.2 Inclusion criteria

Patients who meet all of the following criteria will be included: (1) Patients with migraine meeting the diagnostic criteria of the International Classification of Headache Disorders, 3rd edition (ICHD-3) by the International Headache Society (IHS) in 2018. (2) Participants must be experiencing an active migraine attack at the time of treatment, defined as a VAS score ≥ 4. (3) No preventive treatment with acupuncture or medication has been received in the past week. (4) Age between 18 and 75 years. (5) The individual voluntarily will be signed the informed consent form and will be able to adhere to the treatment regimen. (6) Did not participate in any other clinical studies involving interventions within the past month.

##### 2.1.2.3 Exclusion criteria

Those who meet any of following criteria will be excluded: (1) Patients with severe physical illnesses that prevent them from participating in and cooperating with the trial. (2) Individuals unable to cooperate with physical examination assessments or neuropsychological evaluations due to hearing or visual impairments, aphasia, cognitive disorders, or similar conditions. (3) Patients with concurrent psychiatric disorders. (4) Individuals with poor adherence who were unable to complete clinical observation as required by the protocol. (5) Patients with contraindications to acupuncture, such as pregnant or breastfeeding women.

#### 2.1.3 Withdrawal criteria

Subjects with any of the conditions listed below will be withdrawn: (1) Non-compliance, unwillingness to continue research, or voluntary withdrawal. (2) Serious adverse reactions, complications, or disease progression rendering continued use inappropriate. (3) Failure to adhere to the treatment regimen or insufficient observational data may affect the assessment results.

#### 2.1.4 Sample size calculation

The referenced study reported that after acupuncture treatment, the VAS in the acupuncture group decreased from 5.7 ± 1.4 to 3.3 ± 2.5, representing a mean VAS change of 2.4 points. The VAS in the sham acupuncture group decreased from 5.4 ± 1.3 to 4.7 ± 2.4, reflecting a mean VAS change of 0.7 points (11). The between-group difference in VAS change was therefore 1.7 points. The calculation is performed using the Superiority Tests for the Difference Between Two Means in PASS 15.0 software. With *α* = 0.05 (two-sided), power (1−β) = 0.85, and an anticipated 10%dropout rate, a total of 86 participants (43 per group, 1:1 ratio) are required to detect a statistically significant difference in VAS changes between the two groups.

#### 2.1.5 Randomization

The SPSS 21.0 software will be used to generate a random number table. The participants will be randomly divided into RA group and SA group. The participants’ information will be placed in the envelopes, which will be made of brown, opaque paper. The envelopes will be numbered in order, and the participants will be divided into groups based on the numbers. The factors related to the proteomics and short-term acupuncture response, such as gender, age, education, BMI, underlying diseases, will be recorded.

#### 2.1.6 Blinding

Participants will be blinded. All the participants will be separated into different cubicles to avoid communication during the intervention. Due to the particularity of acupuncture, acupuncturists will not be blinded. All outcome assessors and statistical analysts will be blinded to randomization assignment and intervention.

To assess the success of participant blinding, participants will be asked immediately after the acupuncture session, before they are informed of their group allocation, to indicate which intervention they believe they received: (1) real acupuncture, (2) sham acupuncture, or (3) uncertain. They will also rate their confidence in this judgment on a numerical scale (e.g., 0–10). The distribution of treatment guesses will be reported for each group. The Bang Blinding Index, with its corresponding 95% confidence interval, will be calculated to evaluate the success of blinding. An index close to 0 indicates successful blinding, whereas an index close to 1 indicates failure of blinding.

#### 2.1.7 Interventions

Trained and certified acupuncturists with at least three years of experience will deliver interventions. Subjects in both groups will receive immediate one acupuncture treatment (0.25 × 40 mm, Hwato brand, Suzhou Medical Supplies Factory Co. LTD, Suzhou, China).

##### 2.1.7.1 Basic treatment

Prior to formal intervention, researchers will document participants’ clinical characteristics, including gender, age, allergy history, marital and reproductive history, family history, smoking and alcohol consumption history, past medical history, medication history, and other relevant clinical data. All participants will receive basic treatment, health education, and lifestyle management. They will learn to avoid triggers, get enough sleep, eat regularly, exercise regularly, and learn relaxation techniques and stress management. The application of drugs is according to the Chinese Migraine Diagnosis and Treatment Guidelines (2022) and the Chinese Migraine Integrated Traditional and Western Medicine Prevention and Treatment Guidelines (2022) (1, 12). Acupuncture treatment must be administered within 4 h of attack onset.

To minimize confounding of the 24-hour VAS assessment, participants will be instructed to refrain from taking any rescue medication during the 24 h following acupuncture unless the headache becomes unbearable. If rescue medication is required, flunarizine hydrochloride capsules will be administered, and the dosage, time of administration, and reason for use will be strictly recorded in the case report form. Participants who require rescue medication within 24 h post-treatment will be included in the intention-to-treat (ITT) analysis, with their VAS scores at the time of medication use carried forward for the 24-hour assessment (last observation carried forward, LOCF). A sensitivity analysis excluding participants who used rescue medication will also be performed to evaluate the robustness of the primary findings.

##### 2.1.7.2 Acupuncture group

Patients in the RA group will receive acupuncture on the Baihui (GV20), Sishencong (EX-HN1), Shuigou (GV26), bilateral fengchi (GB20), shuaigu (GB8), taiyang (EX-HN5), taichong (LR3), neiguan (PC6), and shenmen (HT7). The locations of acupoints are shown in Table 1, Figure 2.

**Table 1:** Allocation of acupoints and sham-acupoints.

Acupoints	Real Acupoints Location	Sham Acupoints Location
GV26*(Shuigou)*	On the face, the intersection of the upper 1/3 and the lower 2/3 of the philtrum.	On the face, about 1 cun (≈25 mm) lateral to GV26*,* straight up from the corner of the mouth.
PC6*(Neiguan)*	In the anterior area of the forearm, between the palmar longus tendon and the flexor carpi radialis tendon, and 2 cun (≈ 50 mm) above the transverse striae of the volar side of the wrist.	On the palmar side of the forearm, 2 cun (≈ 50 mm) above the wrist transverse crease, horizontally between the radial wrist flexor tendon and the brachioradialis tendon, 1 cun (≈25 mm) lateral to PC6.
HT7*(Shenmen)*	At the ulnar end of the anterior wrist crease, in the depression radial to the flexor carpi ulnaris tendon.	On the palmar side of the wrist, 0.5 cun (≈12.5 mm) lateral to the HT7.
GB8*(Shuaigu)*	On the head, 1.5 cun (≈40 mm)directly above the apex of the ear and into the hairline	Located on the head, 1 cun (≈25 mm) horizontally lateral to GB8 at the same horizontal level along the hairline.
EX-HN5*(Taiyang)*	In the depression about one transverse finger posterior to the midpoint between the lateral end of the eyebrow and the outer canthus.	0.5 cun (≈12.5 mm) horizontally lateral to EX-HN 5, at the anterior edge of the preauricular hairline.
GB20 *(Fengchi)*	In the posterior region of the neck, under the occiput, in the depression between the upper part of the sternocleidomastoid muscle and the upper part of the trapezius muscle.	1 cun (≈25 mm) directly below GB20.
LR3 *(Taichong)*	On the dorsum of the foot, between the first and second metatarsal bones, in the depression anterior to their bases.	Midpoint between *LR3* and ST41.

**Figure 2:**
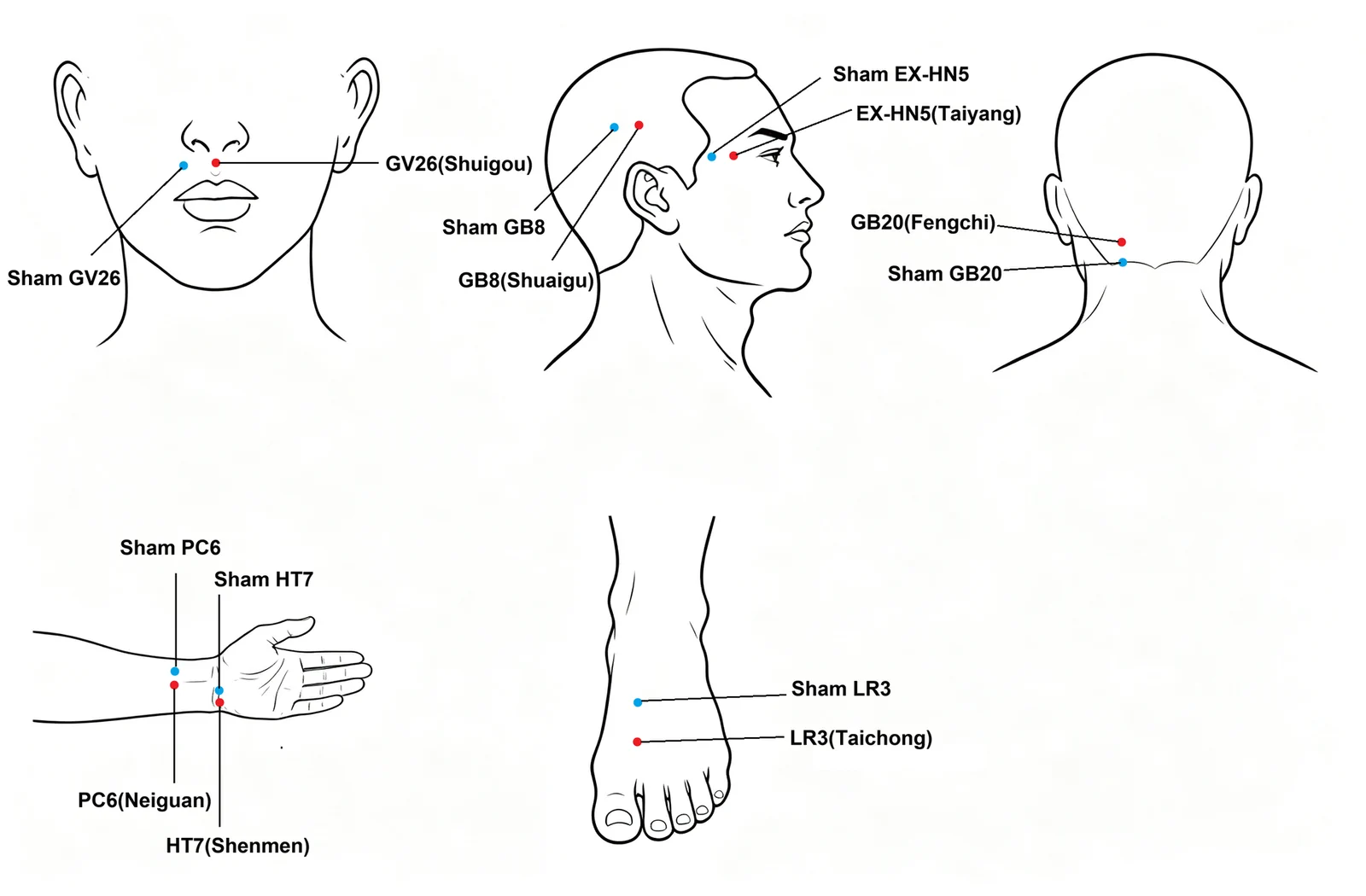
Location of acupoints and sham-acupoints. The inserted depth of acupoints are various: GV20 at a depth of 0.5-1.0 cun (≈ 15–25 mm), PC6 at a depth of 0.5-1.0 cun (≈ 15–25 mm), GV26, EX-HN1, EX-HN5, and HT7 at a depth of 0.3-0.5 cun (≈ 9–15 mm), GB20 at a depth of 0.8-1.2 cun (≈ 25–30 mm), GB8 and LR3 at a depth of 0.5-0.8 cun (≈ 15–20 mm). After needles enter the skin, using lifting-thrusting, twirling-rotating, and sparrow pecking-like manipulation to obtain the sensation of “deqi”. All needles will be retained in the acupoints for 30 min.

Additionally, select acupoints based on the location of the patient’s headache:

Yangming Meridian Headache: Primarily pain in the forehead, brow ridge, and nasal root. Combine with Touwei (ST8), Yintang (EX-HN3), and Neiting (ST44).

Shaoyang Meridian Headache: Primarily pain around the sides of the head and ears. Combine with Waiguan (SJ5) and Zulinqi (GB41).

Taiyang Meridian Headache: Pain in the occipital region or extending to the nape. Combine with Tianzhu (GB10), Houxi (SI3), and Shenmai (BL62).

Jueyin Meridian Headache: Pain concentrated at the vertex or extending to both eyes. Combine with Meichong (EX-HN5), Kunlun (BL60), Shenting (EX-HN3), and Yintang (EX-HN3).

The complete list of syndrome-specific acupoints with their corresponding locations is provided in Supplementary Table 1.

Importantly, Traditional Chinese Medicine meridian pattern differentiation is used solely for individualized acupoint selection within the real acupuncture group and does not serve as an inclusion or diagnostic criterion. The diagnosis of migraine is established exclusively based on ICHD-3 criteria.

##### 2.1.7.3 Sham acupuncture group

The blinding design of the non-acupoint shallow needling method will be chosen (13–15). This method has been used in many published high-quality randomized controlled trials (16–18). The SA group will receive a superficial skin penetration at sham acupoints without manipulation for “deqi”. Sham acupoints are located beside the meridians and anatomical locations associated with Migraine.

#### 2.1.8 Outcome measures

##### 2.1.8.1 Primary outcome measure

The primary outcome is the between-group difference in the change in visual analog scale (VAS) scores from baseline to 24 h post-treatment.

##### 2.1.8.2 Secondary outcome measures

Secondary outcomes include assessment of the change in VAS score at 72 h and 1 week post-acupuncture; Hamilton Anxiety Scale/Hamilton Depression Scale (HAMA/HAMD) at 1 week post-acupuncture; clinical response rates at 24 h, 72 h, and 1 week post-acupuncture; the number of migraine attacks, attack frequency, and attack duration at 24 h, 72 h, and 1 week post-acupuncture.

The efficacy criteria were based on the Guiding Principles for Clinical Research of New Chinese Medicines (2002 edition). The specific definitions are as follows:

Clinical cured: Symptoms disappear with no recurrence within 1 week after the end of treatment.

Markedly effective: Efficacy index > 50%.

Effective: Efficacy index between 20% and 50%.

Ineffective: Efficacy index ≤ 20%.

Note: The efficacy index is calculated as [(VAS _baseline_− VAS _24h_)/VAS_baseline_] × 100%. The total effective rate is defined as the proportion of patients achieving clinical cured, markedly effective, or effective outcomes out of the total number of cases.

### 2.2 Study II: exploration of protein biomarkers for the efficacy of acupuncture intervention in acute migraine

#### 2.2.1 Research participants

Patients enrolled in Study Ⅰ.

#### 2.2.2 Inclusion and exclusion criteria criteria

Inclusion and exclusion criteria will be the same as Study Ⅰ.

#### 2.2.3 Basis for grouping

The efficacy of acupuncture was evaluated in patients after acupuncture treatment. The difference in Visual Analogue Scale (VAS) scores between the two groups at 24 h after acupuncture was taken as the basis for evaluating the clinical efficacy of acupuncture for acute migraine. Treatment response will be evaluated using the absolute change in VAS from baseline to 24 h after acupuncture, calculated as *Δ*VAS = VAS _baseline_−VAS _24h)_, with a larger positive value indicating greater improvement. All participants in the real acupuncture group will be ranked according to *Δ*VAS, from the greatest improvement to the least improvement or worsening. The 10 participants with the largest reductions in VAS will be assigned to the high-response group, whereas the 10 participants with the smallest reductions, no change, or increases in VAS will be assigned to the low-response group. If tied values occur at the selection boundary, the required participants will be selected using a prespecified computer-generated random procedure conducted by an independent statistician.

Baseline clinical information and plasma samples will be collected from all participants in the real acupuncture group before acupuncture. The extreme-response groups will be determined only after completion of the 24-hour VAS assessment. The selected baseline plasma samples will subsequently undergo DIA proteomic profiling. Plasma samples from the remaining participants will be stored for future confirmatory analyses.

#### 2.2.4 Proteomic data analysis

Between Group A and Group B, the raw protein quantification matrix will be initially subjected to data cleaning and preprocessing. Contaminant proteins will be removed, and proteins with a missing value ratio > 80% across all samples will be excluded. Subsequently, log₂-transformation will be applied to improve the data distribution characteristics. For the missing values, a simulated imputation method based on mass spectrometry detection characteristics will be adopted: missing values will be imputed using a normal distribution model with a mean shifted downward by 1.8 standard deviations (SD) and a width of 0.25 SD. Proteins with a missing value ratio exceeding 50% in both groups will be further excluded, and high-quality data will be retained for subsequent analysis.

Differential protein analysis will be performed: differential expression analysis will be conducted in the R environment (version 4.2.2) using the limma and DEP packages. Strict screening criteria will be set: |log₂FC| > 0.585(equivalent to FC > 1.5 or FC < 1/1.5) and false discovery rate (FDR) adjusted *p*-value via the Benjamini-Hochberg method < 0.05. Potential differentially expressed proteins will be screened by proteomic analysis.

#### 2.2.5 Plasma indicator collection time

Before acupuncture at enrollment.

#### 2.2.6 Plasma collection

(1) All subjects will be instructed to maintain a light diet one day before blood collection and fast for at least 8 h prior to venipuncture. A total of 3–5 mL cubital venous blood will be collected using EDTA anticoagulant tubes. (2) Plasma separation: Blood samples will be centrifuged at 3000 rpm for 20 min at 4 °C, and the supernatant will be collected. (3) Blood sample storage: Within 30 min after blood collection, the supernatant (plasma) will be aliquoted into 5 cryovials with screw caps at 0.2 mL per tube and properly labeled. All samples will be stored in a refrigerator at −80 °C. Relevant sample information and storage locations will be recorded in detail.

#### 2.2.7 Plasma protein enrichment and digestion

Twenty baseline plasma samples will be included in the exploratory DIA proteomic analysis, comprising 10 samples from participants with relatively high short-term responses to acupuncture and 10 samples from participants with relatively low responses. For each participant, 20 μL of plasma will be used for protein enrichment.

Each plasma sample will be diluted with loading buffer containing 10 mM Tris-HCl, 1 mM EDTA, 150 mM KCl, and 0.05% CHAPS and mixed thoroughly with 1 mg of superparamagnetic iron-oxide nanoparticles. The mixture will be incubated for 1 h at 37 °C. The magnetic beads will then be washed twice with the loading buffer containing 0.05% CHAPS and once with CHAPS-free washing buffer containing 10 mM Tris-HCl, 1 mM EDTA, and 150 mM KCl. The protein-enriched magnetic beads will be collected using a magnetic separator, and the supernatant will be discarded.

The protein-enriched beads will be resuspended in lysis buffer containing 1% sodium deoxycholate, 100 mM Tris-HCl at pH 8.5, 10 mM tris (2-carboxyethyl)phosphine, and 40 mM chloroacetamide. Reduction and alkylation will be performed simultaneously by incubation for 30 min at 60 °C. An equal volume of double-distilled water will subsequently be added to reduce the sodium deoxycholate concentration to below 0.5%.

Proteins will be digested overnight at 37 °C with shaking using 1 μg of sequencing-grade trypsin per sample. Digestion will be terminated by adding 0.1% trifluoroacetic acid. The resulting peptides will be desalted using styrene-divinylbenzene reversed-phase sulfonate cartridges, dried under vacuum, and stored at - 20 °C. Before LC-MS/MS analysis, the dried peptides will be reconstituted in 0.1% trifluoroacetic acid, and the peptide concentration will be measured.

#### 2.2.8 Nano-LC separation and diaPASEF acquisition

Peptide samples will be analyzed using an UltiMate 3000 RSLCnano liquid-chromatography system (Thermo Fisher Scientific) coupled to a timsTOF Pro mass spectrometer (Bruker Daltonics). An appropriate amount of peptide from each sample will be loaded using an autosampler onto a C18 trap column (75 µm × 2 cm, 3 µm particle size, 100 Å pore size; Thermo Fisher Scientific). Peptides will subsequently be separated using a C18 analytical column (75 µm × 25 cm, 1.6 μm particle size, 100 Å pore size; IonOpticks).

Mobile phase A will consist of 0.1% formic acid in water, and mobile phase B will consist of 0.1% formic acid in acetonitrile. Chromatographic separation will be performed at a flow rate of 300 nL/min using a predefined gradient.

Mass-spectrometric data will be acquired in data-independent parallel accumulation-serial fragmentation mode. The capillary voltage will be set to 1,400 V. The MS1 and MS2 scan ranges will be set to m/z 100–1700. The inverse reduced ion-mobility range will be 1/K0 = 0.6 Vs/cm^2. Both the accumulation time and ramp time will be set to 100 ms.

The diaPASEF isolation-window scheme will be configured using timsControl software according to the two-dimensional distribution of precursor mass-to-charge ratio and ion mobility. Collision energy will be linearly ramped from 20 eV at 1/K0 = 0.6 Vs/cm^2 to 59 eV at 1/K0 = 0.6 Vs/cm^2.

#### 2.2.9 Database searching and protein quantification

The raw diaPASEF data will be processed using DIA-NN software, version 1.8.1, in library-free mode. The human protein-sequence database downloaded from UniProt on 19 June 2023 will be used for database searching. The theoretical spectral library will be generated by enabling the Precursor ion generation option in DIA-NN.

Trypsin/P will be specified as the proteolytic enzyme, with up to two missed cleavages permitted. Carbamidomethylation of cysteine will be defined as a fixed modification, whereas oxidation of methionine and protein N-terminal acetylation will be defined as variable modifications. The MS1 and MS2 mass tolerances will both be set to 15 ppm. Match between runs and heuristic protein inference will be enabled. The false discovery rate will be controlled at 1%. Protein abundance will be quantified and normalized using the MaxLFQ algorithm.

### 2.3 Study Ⅲ construction of efficacy prediction model based on acupuncture intervention, plasma proteins and clinical outcome in acute migraine patients

#### 2.3.1 Research participants

Patients enrolled in Study Ⅰ.

#### 2.3.2 Inclusion and exclusion criteria criteria

Inclusion and exclusion criteria will be the same as Study Ⅰ.

#### 2.3.3 Data collection

Including general information, laboratory tests, risk factor screening, clinical scales, and other related data: (1) General information: Includes age, gender, body mass index, disease duration, blood pressure, blood glucose, medication regimen (5-HT receptor agonists, nonsteroidal anti-inflammatory drugs, *β*-blockers, calcium channel blockers, anti-anxiety and antidepressant drugs, etc.), clinical scale scores at enrollment, etc. (2) Laboratory tests: Including coagulation function, blood routine, liver function, renal function, etc. within one week before enrollment. (3) Risk factors: comorbidities, smoking and drinking status, sleep status, personal history, and family history of migraines, etc. (4) Imaging examination: Existing cranial MRI results of the subjects will be collected, and the presence or absence of cerebral infarction, cerebral hemorrhage, cerebral white matter lesions, cerebral arteriosclerosis, cerebral artery stenosis or occlusion will be recorded.

#### 2.3.4 Exploratory model development and internal evaluation

An exploratory proof-of-concept model will be developed using the 20 selected participants (10 high-responders and 10 low-responders) from the real acupuncture group, for whom complete baseline clinical data and proteomic profiles will be available. The modeling cohort will therefore be identical to the proteomic cohort described in Study II. The purpose of this analysis is to investigate whether a small set of baseline clinical and protein features may contain preliminary predictive information regarding short-term acupuncture response; it is not intended to establish a clinically deployable or validated prediction model.

Given the limited sample size, all 20 participants will be retained for exploratory model development, and no 75%/25% training-test split will be performed. Random Forest, XGBoost, support vector machine, and other complex machine-learning algorithms will not be compared. Candidate predictors will be restricted to a small number of clinical variables and proteins selected on the basis of prior biological evidence and the exploratory findings of Study II. A parsimonious penalized logistic regression model using LASSO or elastic net regularization will be considered.

To minimize information leakage, all protein feature selection steps will be repeated independently within each training fold of the cross-validation procedure. This includes the differential-protein screening step described in Section 2.2.4. Specifically, within each training fold, we will re-identify differentially expressed proteins using only the training samples, applying the same criteria as in Study II (|log₂FC| > 0.585, FDR-adjusted *p* < 0.05). These within-fold selected proteins, along with a small number of prespecified clinical variables, will then be used to fit the penalized logistic regression model in that fold. No protein-level information from the validation fold will be used in any step of the feature-selection or model-development process.

Nested leave-one-out cross-validation will be used to generate internally cross-validated predictions. To minimize information leakage, normalization, missing-data handling, candidate-feature selection, model fitting, and hyperparameter tuning will all be performed within each training fold. Model discrimination and overall prediction error will be summarized using the cross-validated AUC and Brier score with corresponding confidence intervals, and predictor-selection stability across resampling iterations will be reported. These estimates will be interpreted cautiously as exploratory and hypothesis-generating; independent external validation in a substantially larger cohort will be required before any clinical application.

### 2.4 Data management

Clinical data will be managed with the clinical research data management system of the Zhejiang Provincial Hospital of Traditional Chinese Medicine.

### 2.5 Statistical methods

Statistical analyses will be performed using SPSS 26.0 software. All outcome measures will be analyzed based on the intention-to-treat (ITT) principle. For the primary outcome, an independent-samples t-test will be used to compare the change in VAS scores from baseline to 24 h between the real acupuncture and sham acupuncture groups. Continuous variables will be assessed for normality and homogeneity of variance. Normally distributed continuous data will be presented as mean ± standard deviation (SD), whereas non-normally distributed data will be expressed as median and interquartile range, denoted as Q2 (Q1, Q3). Categorical variables will be described using frequencies, proportions, and rates. For normally distributed continuous variables, within-group comparisons before and after the intervention will be conducted using paired-samples t-tests; for non-normally distributed data, the Wilcoxon signed-rank test will be applied. In cases involving multiple paired samples within groups, repeated measures analysis of variance (ANOVA) will be employed for normally distributed continuous data, while the Friedman test will be used for non-normally distributed continuous data. The absolute change in VAS from baseline to 24 h will be analyzed as a continuous outcome in all participants in the real acupuncture group. Descriptive statistics will be used to summarize the distribution of *Δ*VAS. General vital signs, relevant laboratory parameters, medication usage, adverse events, and adverse reactions will be summarized using descriptive statistics.

### 2.6 Patient safety

Any Adverse Events (AEs) or Serious Adverse Events (SAEs) that occurred during the intervention, whether or not related to the intervention, will be recorded in detail. Researchers will judge the relevance of AEs/SAEs and treatment based on information such as the cause, time, and process. Common acupuncture-related AEs include fainting, stuck needles, broken needles, bent needles, bleeding, edema, and other minor AEs will be treated by the attending acupuncturist immediately. All SAEs must be reported to the Ethics Committee within 24 h.

### 2.7 Dissemination

The study will comply with the declaration of Helsinki principles (World Medical Association version, 2013). All participants will voluntarily sign an informed consent form before participating in this trial. The study has been registered at the Chinese Clinical Trial Registry (ITMCTR2026000359).

## 3 Discussion

The present study adopts a prospective, randomized, single-blind design to evaluate the efficacy of a single acupuncture session for acute migraine and to explore baseline protein biomarkers potentially associated with short-term treatment response. The integration of clinical and proteomic information may help identify candidate predictors for subsequent investigation. Given the small proteomic cohort, the proposed modeling analysis is explicitly exploratory and is intended to assess feasibility and generate hypotheses rather than to produce a validated clinical prediction or decision-support tool.

Migraine is a highly prevalent and disabling neurological disorder characterized by recurrent episodes, which imposes a substantial global public health burden (19). Current Western pharmacological interventions primarily target single molecular pathways, often yielding suboptimal therapeutic effects (3). Notably, approximately 40% of migraine patients develop medication-overuse headache, which contributes to recurrent headache exacerbations and forms a refractory pathological cycle. Acupuncture is one of the most common alternative and complementary therapies worldwide, and has been recommended for the treatment of musculoskeletal, connective tissue, and neurological disorders (20). Acupuncture was officially recommended by the World Health Organization as a therapy for migraines in 1979. In recent years, a large amount of domestic and international research evidence has shown that acupuncture has significant advantages in treating migraines (21–23). Multiple guidelines recommend that acupuncture can be used as both acute pain relief treatment and preventive treatment during the remission period (1, 12). This study is designed to predict short-term (1-week) treatment response, while long-term preventive effects will need to be explored in future studies.

In this study, clinical outcomes were comprehensively assessed using multiple validated indicators, including the VAS score, HAMA and HAMD scales, and migraine attack status at the 1-week follow-up (including attack incidence, frequency, and duration). The VAS scale, a well-established quantitative tool for subjective symptom evaluation, was adopted to precisely quantify headache severity. Compared with categorical grading scales, VAS converts discrete clinical scores into continuous data, which significantly improves data sensitivity and statistical reliability, rendering it suitable for evaluating dynamic changes in pain symptoms. Accumulating evidence indicates that the prevalence of anxiety disorders and depression is significantly higher in patients with migraine compared to the general population, and these comorbidities are closely associated with greater headache severity, prolonged disease course, and reduced quality of life (24, 25). The HAMA and HAMD scales, with robust reliability, validity and reproducibility, allow accurate quantification of emotional disturbances and enable a comprehensive evaluation of the physical and mental status of migraine patients, thus ensuring a multidimensional and objective assessment of acupuncture intervention effects.

Proteomics technology enables the systematic profiling of global protein expression alterations in pathological states, which is an effective strategy for screening disease-specific biomarkers and exploring molecular mechanisms of therapeutic interventions. To date, proteomic analysis has been widely applied in the research of various refractory diseases. In oncology, differential protein profiling has been successfully used for molecular subtyping of ovarian cancers, greatly promoting the development of precision tumor therapy (26). In neurological research, distinct protein biomarkers derived from brain tissue have been identified for Alzheimer's disease, solving the clinical dilemma of delayed diagnosis due to the insidious onset of neurodegenerative disorders (27, 28).

Prior studies exploring the effects of acupuncture on migraine have largely relied on single-index detection, which fails to fully reflect the multi-target and holistic regulatory characteristics of acupuncture treatment. In contrast, proteomic profiling can systematically characterize global protein expression changes after acupuncture intervention, overcoming the limitations of conventional single-mechanism research. Previous study have shown that a large number of proteins are abnormally expressed in the plasma and other samples of migraine patients (29). These proteins are closely associated with cellular damage, oxidative stress and inflammation. Accordingly, proteomic approaches can be used to distinguish between migraine episodes and headache-free status. Acupuncture intervention can effectively regulate and reverse the abnormal expression of proteins, and remodel the disturbed protein profiles in the body, thereby reducing the frequency and duration of migraine attacks. The candidate proteins identified in this study will reflect baseline differences between patients with relatively high and low short-term responses to acupuncture. They should not be interpreted as migraine-specific biomarkers or as evidence that acupuncture normalizes an abnormal proteomic profile. Determining disease normalization would require comparisons with matched healthy controls and longitudinal assessment of protein expression before and after acupuncture.

Building on previous mechanistic research, the present study will explore whether baseline proteomic signatures and clinical characteristics differ between participants with high and low short-term responses to acupuncture. The exploratory integration of these data may prioritize a small number of candidate biomarkers for future confirmatory studies. Any internally cross-validated performance observed in this small cohort will be interpreted as preliminary and will not establish clinical utility. A substantially larger, prospectively recruited cohort and an independent external-validation dataset will be required to develop a stable model, evaluate calibration and clinical utility, and determine whether the candidate biomarkers can support individualized treatment decisions.

### 3.1 limitations

Several limitations of this study should be acknowledged. First, this study evaluates the immediate efficacy of acupuncture for acute migraine attacks, with a follow-up period of only one week; therefore, the findings cannot be extrapolated to the long-term preventive effects of acupuncture. Second, the proteomic analysis compares baseline plasma profiles between responders and non-responders within the real acupuncture group and does not include a matched healthy control cohort or longitudinal post-treatment proteomic measurements. Consequently, the study cannot determine whether the identified candidate proteins represent migraine-related abnormalities, normal interindividual physiological variation, or treatment-induced normalization. The identified proteins will therefore be interpreted only as exploratory baseline biomarkers associated with short-term acupuncture response. Future studies should include matched healthy controls and repeated plasma sampling before and after acupuncture to clarify their biological and clinical significance. Third, the proteomic and modeling cohort includes only 20 participants selected from the real acupuncture group. This sample size is insufficient for reliable development or validation of a multivariable clinical prediction model, particularly when the number of potential protein features is large. Although a parsimonious penalized model and nested leave-one-out cross-validation will be used to reduce and quantify overfitting, internal resampling cannot overcome the fundamental statistical uncertainty arising from the small sample. The resulting model and its performance estimates will therefore be considered strictly exploratory and hypothesis-generating, not evidence of clinical utility. Fourth, the DIA proteomic analysis adopts an extreme-phenotype sampling strategy by selecting participants from the two ends of the continuous VAS-response distribution. Although this approach may improve the efficiency of preliminary biomarker discovery in a resource-limited exploratory study, it may exaggerate apparent between-group differences and does not represent the full spectrum of treatment response. Accordingly, the proteomic findings will be considered hypothesis-generating and will require confirmation using stored baseline plasma samples from a larger cohort in which VAS change is analyzed continuously. Fifth, this study does not assess longer-term outcomes such as migraine-related disability, quality of life, or medication consumption. Finally, the single-center design may limit generalizability. Future studies should recruit a substantially larger cohort representing the full range of responses and perform independent external validation before the model is considered for clinical application.
